# Multiple Facets of Venous Thrombosis

**DOI:** 10.3390/ijms22083853

**Published:** 2021-04-08

**Authors:** Alexander Brill

**Affiliations:** 1 Institute of Cardiovascular Sciences, College of Medical and Dental Sciences, University of Birmingham, Birmingham B152 TT, UK; A.Brill@bham.ac.uk; 2 Centre of Membrane Proteins and Receptors (COMPARE), Universities of Birmingham and Nottingham, The Midlands NG7 2UH, UK; 3 Department of Pathophysiology, Sechenov First Moscow State Medical University (Sechenov University), 119991 Moscow, Russia

Venous thromboembolism, a complex disease combining deep vein thrombosis (DVT) and its most dangerous complication, pulmonary embolism (PE), strikes millions of people worldwide. For example, in the United States only, DVT develops in up to 600,000 individuals annually, and 100,000–180,000 deaths from VTE are estimated each year [1]. The disease is considered an emergency condition and requires immediate medical attention. Various risk factors predispose to DVT, such as trauma, cancer, or genetic mutations promoting blood hypercoagulability. Venous thrombosis can also develop in people after major surgery leading to prolonged bed-ridden position. In the latter case, blood stagnancy in veins is considered one of the major triggers of thrombosis. Blood flow disturbance is one of three components of Virchow’s triad, which also includes hypercoagulability of the blood and procoagulant state of the vessel wall. Blood stasis is recapitulated by complete or partial ligation of the inferior vena cava in most widely used animal (usually mouse or rat) models of DVT [2].

Studies in the field of venous thrombosis cover several aspects of the disease. Discovery of the mechanisms of thrombosis initiation and propagation could help prevent its development in individuals at risk. Exploration of the routes of thrombus resolution is essential to reduce deleterious effects of the formed clot. Finally, search for specific biomarkers is necessary for both verification of the diagnosis and, importantly, prediction of the probability of thrombosis. A review by Anghel et al. in this issue Journal presents a set of previously known as well as novel biomarkers of venous thrombosis and discusses their potential benefits and limitations in diagnosis and building the treatments strategy for DVT [3].

Separate mechanisms of DVT in certain pathological conditions are relatively well understood. For example, the role of tissue factor in cancer-related DVT has been established [4]. However, we are still far from full understanding of all factors driving thrombosis in veins or affecting its resolution. In this Special Issue, works from different groups are presented uncovering new aspects of DVT or summarizing existing knowledge on the topic. Whereas the role of the innate immune system in DVT has been reported [5,6], Mukhopadhyay and colleagues demonstrate previously underestimated involvement of the adaptive immune system in thrombus resolution [7]. These authors show that depletion of T-cells unexpectedly reduces the numbers of macrophages in the thrombus and decreases activity of the fibrinolytic system and metalloproteinase-9, involved in thrombus dissolution. The immune cell-dependent mechanisms of thrombus resolution are further explicitly reviewed by Nicklas et al. [8]. These findings identify novel potentially useful targets to fight venous thrombosis.

Venous wall does not contain *vasa vasorum* and receives oxygen and nutrients from the blood inside the vessel. Blood flow stagnancy results in limited supply of oxygen and local hypoxia of both endothelium and subendothelial layers, which leads to formation of reactive oxygen species (ROS) [9]. An important role of ROS (for example, hydrogen peroxide) in venous thrombosis has recently been demonstrated [10]. In this issue, Gutmann and co-authors provide an explicit overview of various aspects of ROS involvement in DVT [11]. Authors discuss the role of ROS in blood clotting and fibrinolysis, involvement of ROS in functioning of blood cells, such as platelets and erythrocytes, ROS impact in formation of neutrophils extracellular traps (NETs) and venous thrombus resolution as well as other effects of ROS relevant to thrombosis in veins.

According to the currently predominant concept, PE develops when a thrombus or its part gets dislodged from the site of initial formation (e.g., deep veins of the leg) and travels all the way to the lungs where it occludes branches of the pulmonary artery. However, clinical evidence suggests that in many cases, clots in the lungs are observed in the absence of primary thrombosis elsewhere. Porembskaya et al. discuss an alternative scenario, in which systemic prothrombotic and proinflammatory milieu could mediate the development of thrombi in lungs in situ independently of DVT [12]. According to authors’ hypothesis, primary pulmonary thrombosis can result from such pathological conditions as trauma, major surgery, chronic obstructive pulmonary disease of sickle cell disease (SCD). Mechanisms of thrombosis is SCD are discussed in detail by Lizarralde-Iragorri and colleagues [13]. Authors provide an overview of prothrombotic shift in SCD including blood stasis and proinflammatory environment. Importantly, existing mouse models of SCD are presented and thoroughly discussed in this review.

The coverage of VTE-related topics would be incomplete without its clinical aspects. Patel et al. present a comprehensive analysis of existing approaches for diagnostics and prediction of VTE [14]. The article discusses diagnostic strategies involving biochemical markers, imaging data, and clinical scores to identify either DVT or PE. A special emphasis is put on diagnosis of the diseases in pregnant versus non-pregnant individuals.

It is known that pulmonary hypertension (PH) is frequently observed and serious complications of PE [15]. A study by Vrigkou and colleagues analyses blood clotting as well as platelet and endothelial status in patients with PH [16]. Authors demonstrate both impaired platelet functions and blood coagulation, accompanied by reduced thrombin generation ability. These defects can probably be attributed to exhausted capacity of both systems due to previous functional overload.

In conclusion, this Special Issue presents new data and state-of-the-art overview of major aspects of venous thrombosis, such as its development, resolution, diagnosis, and potential biomarkers. This information may be useful to both basic scientists and clinicians dealing with this debilitating and life-threatening disease.
